# Bipolar disorder associated with tuberous sclerosis: Chance association or aetiological relationship?

**DOI:** 10.4103/0019-5545.31624

**Published:** 2006

**Authors:** V.K. Chopra, Y. Cintury, V.K. Sinha

**Affiliations:** * Senior Resident; ** Junior Resident; *** Associate Professor

**Keywords:** Tuberous sclerosis, bipolar disorder, secondary mania

## Abstract

Tuberous sclerosis is a rare disorder. Mental retardation, epilepsy, autism and hyperactivity are commonly reported neuropsychiatric disorders associated with tuberous sclerosis. Rarely, other psychiatric disorders such as psychosis, depression and anxiety associated with this condition have been reported in the literature. A case of bipolar disorder associated with tuberous sclerosis with onset of the first manic episode at the age of 7 years is reported. The possibility of tuberous sclerosis as one of the causes of secondary mood disorder in very young children is also discussed.

## INTRODUCTION

Various studies have reported that the incidence of tuberous sclerosis is around 1 in 10 000.^1^ Although several sets of operationalized criteria have been proposed to diagnose the condition, the diagnosis relies upon clinical signs and symptoms. One very commonly used set of criteria was developed by Gomez and revised by Osborne.^2^ According to these criteria, for definitive diagnosis, only one primary feature from the following is required. Classical shagreen patch, retinal hamartomas, multiple subependymal nodules, periungual fibromas, facial angiofibromas (also called adenoma sebaceum) and multiple renal angiomyolipomas. However, only a presumptive diagnosis can be made when at least two secondary features are present. These secondary features include infantile spasms, other kinds of epilepsies, and various kidney, lung, brain and cardiac lesions. Over 95% of patients have one or another form of brain lesions.^3^ The commonest among them are subependymal nodules (SENS) and cortical tubers. Whereas SENS are easily identified on CT scan (especially when calcified), tubers are better identified on MRI.

Up to 62% of patients tuberous sclerosis have some form of epilepsy.^4^ Intellectual and cognitive impairment is present in the range of 38%-64%.^4^,^5^ As early as 1932, Critchley and Earl^6^ mentioned that young children with tuberous sclerosis show some features of autism, but the term autistic behaviour in connection with tuberous sclerosis was first used in 1967. In recent years, many case reports and studies have found that the co-occurrence of tuberous sclerosis with either classical autism or broadly defined pervasive development disorder is fairly common.^7^,^8^ Another commonly found psychiatric disturbance in patients of tuberous sclerosis is hyperactivity.^5^,^8^ Apart from the above, a few reports have described the occurrence of other psychiatric conditions in patients with tuberous sclerosis including psychosis;^9^ prominent sleep disturbance;^10^ anxiety and depression;^11^ Capgrass syndrome.^12^ To our knowledge, till date, only one case report^13^ has described the association of tuberous sclerosis with childhood mania.

## THE CASE

NP, an 18-year-old girl from rural Orissa, reported to the OPD of the Central Institute of Psychiatry with 2 months' history suggestive of talkativeness, decreased sleep, disinhibited behaviour, demanding attitude and suspiciousness. Her past history was suggestive of two manic episodes in 1990 and 1995 which were treated by a private psychiatrist and the patient had reached the premorbid level (details of treatment were not available). There was no contributory family history. The patient was the last of 5 siblings and all the older ones were healthy. She was born by a normal vaginal delivery conducted in the hospital without any prenatal, intranatal or postnatal complications. All motor and speech milestones were within normal limits and the patient went to an ordinary school and was a student of X standard at the time of presentation and her school performance was rated as good.

**Fig 1.**
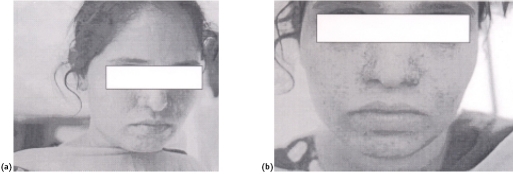
(a and b) Adenoma sebaceum on the face

Physical examination revealed adenoma sebaceum on the face (Fig. 1). Apart from that, there was nothing remarkable. Mental status examination revealed increased psychomotor activity, increased speech, grandiose ideation, cheerful affect and auditory hallucinations.

**Fig 2.**
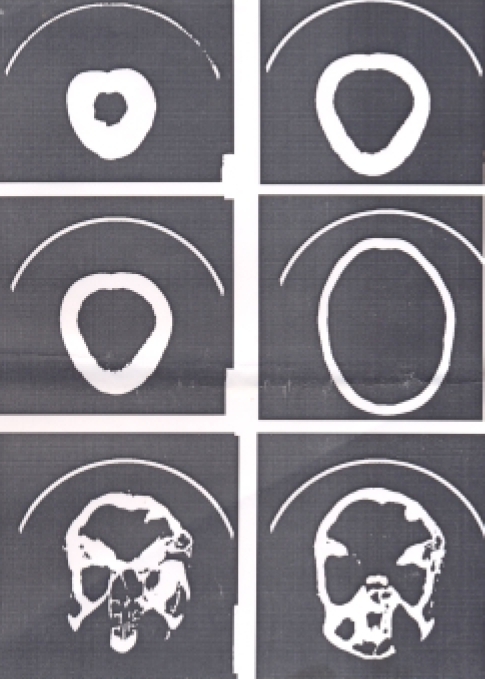
CT scan showing multiple subependymal nodules along the walls of the lateral ventricles. Cortical tubers are seen in the posterior fossa.

A diagnosis of bipolar affective disorder—third episode, currently mania with psychotic features with tuberous sclerosis was made. The patient was put on 3 mg of risperidone and 400 mg of sodium valproate, which was raised to 600 mg per day. She was treated as an outpatient and was doing well on this regimen when seen 2 months after the initial presentation. During follow-up visits, an IQ assessment and a CT scan were done. IQ assessment showed a composite IQ of 102 which showed that she had average intelligence. CT scan findings (Fig. 2) showed multiple subependymal nodules present typically along the walls of the lateral ventricles. Cortical tubers were seen in the posterior fossa. Thus, the physical findings of adenomae sebaceum and CT findings confirmed the diagnosis of tuberous sclerosis. An EEG showed sharp and slow wave discharges on the right side with a preponderance in the frontotemporal region.

## DISCUSSION

Epilepsy, mental retardation, pervasive development disorders and hyperactivity are the neuropsychiatric disorders known to be commonly associated with tuberous sclerosis. It is unknown whether other psychiatric disorders seen in patients of tuberous sclerosis are merely chance associations.^3^ As far as mania and bipolar disorder are concerned, only one case report of a patient with tuberous sclerosis who developed manic symptoms at a very young age has been described.^13^ That patient was a 5-year-old girl with subnormal intelligence, generalized epilepsy and a grossly abnormal EEG with frequent seizure discharges and a well-defined right temporal lobe focus.

As per DSM-IV guidelines,^14^ the diagnosis of mood disorder due to a general medical condition is made when mood disorder is judged to be aetiologically related to the general medical condition. No clear-cut guidelines are described to make this judgement. However, several points which are taken into consideration include the presence of a temporal association between the onset, exacerbation or remission of the general medical condition and that of mood disturbance. Another consideration is the presence of features that are atypical of a primary mood disorder (e.g. atypical age of onset or course or absence of family history). The patient reported by us had her first manic episode at the age of 7 years and there was no family history of mood disorders. As early childhood mania is rare^13^,^15^ and so is tuberous sclerosis, their co-occurrence in the same person may not be just a coincidence.^13^ The patient also had EEG abnormalities in the right frontotemporal region, though clinically no seizures were present. Right-sided brain lesions are known to be associated with manic symptoms as compared to left-sided ones.^16^

Since it is difficult to comment about the possibility of tuberous sclerosis being aetiologically related to bipolar disorder on the basis of a single case report, this is an area for future research. In a patient with tuberous sclerosis presenting with bipolar disorder at a very young age with associated EEG abnormalities and absence of family history, the psychiatric condition may be secondary to the medical condition. The association may not affect the management of the mood disorder, but it is important for parental screening and genetic counselling.^17^
